# Acute transverse myelitis of childhood due to novel coronavirus disease 2019: The first pediatric case report and review of literature

**DOI:** 10.22037/ijcn.v15i1.31579

**Published:** 2021

**Authors:** Habibeh NEJAD BIGLARI, Reza SINAEI, Sara PEZESHKI, Fatemeh KHAJEH HASANI

**Affiliations:** 1 Pediatric Neurology, Neuroscience Research Center, institute of neuropharmacology, Kerman University of Medical Sciences, Kerman, Iran; 2 Pediatrics Rheumatology, Department of Pediatrics, Kerman University of Medical Sciences, Kerman, Iran.; 3 Endocrinology and Metabolism Research Center, Institute of Basic and Clinical Physiology Sciences, Kerman University of Medical Sciences, Kerman, Iran.; 4 Department of Radiology, School of Medicine, Kerman University of Medical Sciences, Kerman, Iran

**Keywords:** Transverse myelitis, neuromyelitis optica, COVID-19

## Abstract

The global coronavirus disease 2019 (COVID-19) pandemic appears to have some streaks of severity in pediatrics. These streaks include variable signs of respiratory distress, a new entity called multi-system inflammatory syndrome, and some evidences of neurological symptoms involving both central and peripheral nervous systems. Here, we described the first pediatric patient with COVID-19 who presented with acute transverse myelitis. An 11-year-old otherwise healthy girl presented to our clinic with acute onset of lower limbs paresis, urinary and fecal retention, alongside epigastric pain, and fever for 3 days. A neurological examination revealed a severe flaccid paraplegia in her lower limbs associated with a sensory level at T5. She was evaluated systematically for all probable causes of her symptoms, and finally, due to having a positive nasopharyngeal PCR test, she was considered to suffer from post-COVID-19 transverse myelitis. She underwent intravenous-immunoglobulin, methylprednisolone pulse, and other supportive cares without obvious results. Therefore, she underwent seven sessions of plasma exchange with little effects on muscle strength. The focal inflammation and injury of the spinal cord, otherwise known as transverse myelitis, have a wide array of potential etiologies. Transverse myelitis has been well documented to be the result of viral and bacterial infections. We believe our patient was not involved in a cytokine storm status due to good CRP, IL-6 and Ferritin levels. Albeit, we cannot certainly consider the patient to have a direct viral impactor involved in a late immunity process. To our knowledge, this is the first report of TM in the field of pediatrics occurred after COVID-19. Thus, this is critical to note that children can present with some severe types of COVID-19.

## Introduction

The global COVID-19 pandemic initially appeared to have milder courses in pediatrics. Since the last few weeks, there have been some streaks of severity in pediatric age reports, characterized by persistent fever, shock, myocarditis, and variable signs of a new entity called multi-system inflammatory syndrome (MIS-C) (1). Moreover, children with COVID-19-related MIS-C may experience neurological symptoms that involve both the central and peripheral nervous systems (CNS and PNS). In a case series of four pediatric patients with COVID-19 and neurological-related symptoms, encephalopathy, headache, brain stem and cerebellar signs, muscle weakness, and reduced reflexes were reported. All the patients had signal changes in the splenium of corpus callosum on neuroimaging in the absence of respiratory symptoms (2).

Since severe acute respiratory syndrome coronavirus 2 (SARS-CoV-2)enters cells via the angiotensin-converting enzyme 2 (ACE-2) receptor, and given that ACE-2 is expressed in both neurons and glial cells, a direct viral invasion of CNS may be a probable mechanism for neurological manifestations of COVID-19 (3). However, only vascular cells and not neurons express ACE-2 in the brain; thus this potentially contributes to dissemination of virus into the brain by blood circulation (4). Albeit, an immune-mediated neuronal syndrome has been recently proposed in some adult neurological reports (5). Here, we reported an 11-year-old girl who presented with new-onset neurological symptoms and neurological imaging findings identic with the diagnosis of acute transverse myelitis associated with COVID-19.

## Case report

**Fig. 1:**
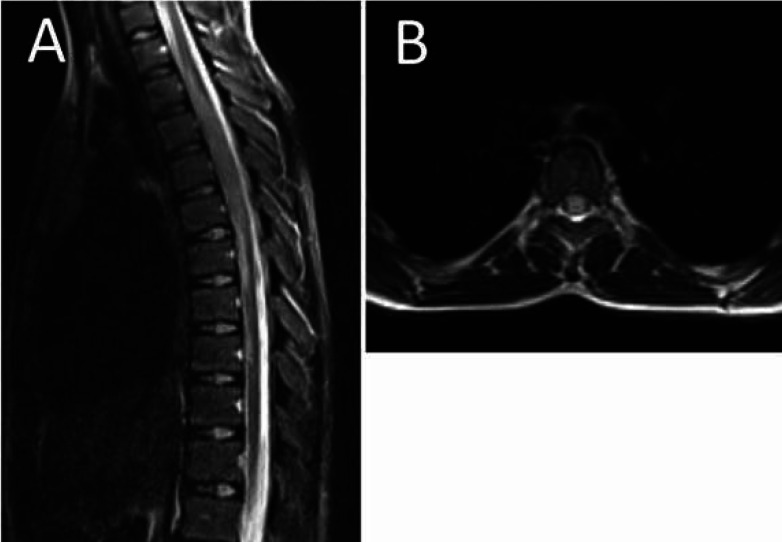
(A) The sagittal T2 weighted image of the thoracic spinal cord showing high signal intensity at T3-T9 segments of the spinal cord associated with spinal cord swelling at T3-T6 segments. (B) The axial T2 weighted image of thoracic spine showing spinal cord swelling and a central high signal area observed in the both halves of the spinal cord

**Fig. 2:**
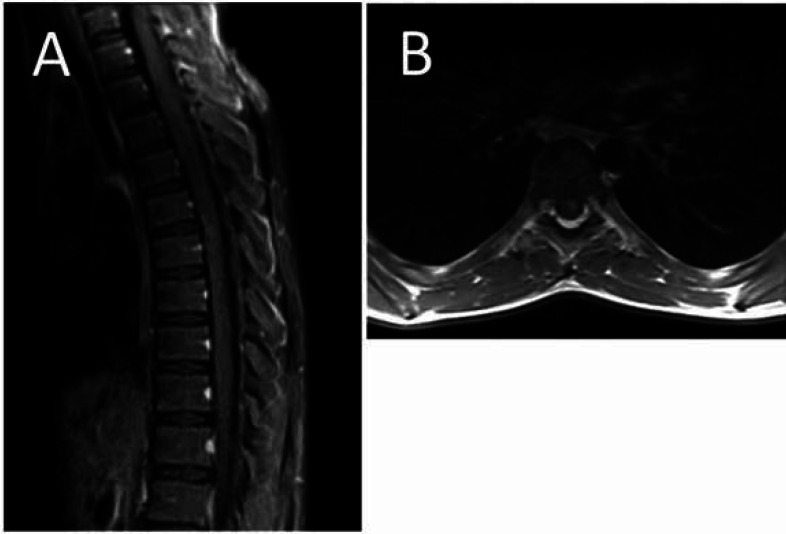
The sagittal (A) and axial (B) post-contrast T1 weighted image showing mild heterogeneous patchy enhancement in the involved area

An 11-year-old otherwise healthy girl presented to our clinic with acute onset of lower limbs paresis, weakness, and urinary and fecal retention. She had also experienced epigastric pain and fever alongside her neurological symptoms for 3 days. She had no history of other respiratory and gastrointestinal manifestations, previous medical illnesses, and medication consumption. At the time of admission, a neurological examination revealed a severe flaccid paraplegia in proximal of her lower limbs associated with a positive Lhermitte’s sign (electric shock-like sensation down the back triggered by forward bending of head), and a sensory level at T5. The upper limb force was near normal, and the lower limb force was 0-5. The Babinski’s sign had a natural response bilaterally. Moreover, deep tendon reflexes and the abdominal reflex were absent. In addition, the level of consciousness, cognition, and cranial nerves was unaffected, and the bulbar sign was intact. The patient underwent the Foley catheter insertion procedure due to urinary retention, and she was evaluated systematically for all probable causes of her dilemma. Initial labs revealed a positive nasopharyngeal swab for SARS-CoV-2. The laboratory results showed a normal white blood cell count of 7.7^3/UL (4.0-10.0^3/UL), with 70% neutrophil and 26% lymphocyte. Hemoglobin, MCV, and platelet count were 11.5 gr/dl (12-15.5), 76.5 fL (80-100), and 350^3/mL (150-450^3/mL), respectively. C-reactive protein (CRP) was in normal range (1mg/L), while the erythrocyte sediment rate was minimally higher (24 mm/h) than normal (<12). No obvious changes were detected in electrolytes. The interleukine-6 level with the ELISA method was 12.8 pg/ml (14±6). D-Dimer, coagulation profile PT (12 sec), PTT (32 sec), and INR (1.0) were all in normal ranges. SGOT, SGPT, and creatinine were 51 U/L, 17 U/L, and 0.4 mg/dl, respectively, and they were all in normal ranges. Certain phosphokinase was 3144 U/L (24-170), and lactate dehydrogenase was 368 (<580). The patient was evaluated serologically for some other potential viruses including EBV, CMV, and influenza A and B) as well as bacteria (mycoplasma pneumonia), and the results were negative. The auto-immune rheumatological screening revealed negative results regarding anti-nuclear antibody, complement level, anti-cytoplasmic antibodies, angiotensin converting enzyme, rheumatoid factor, and anti-phospholipid antibodies. Although, the cerebrospinal fluid (CSF) had no obvious finding in analysis and culture, antibodies and viral PCR of CSF fluid were not performed during hospitalization. In electromyoneurography, F and H responses were unobtainable in the patient’s lower limbs. Echocardiography showed PFO (5-6 mm) without any evidence of embolism or functional effects. The chest x-ray was normal with no evidence of consolidation, cardiomegaly, and pleural effusion. A Gadolinium-enhanced magnetic resonance imaging of the brain and whole spine was performed, revealing no changes in the brain but a high signal intensity at T3-T9 segments of the spinal cord associated with spinal cord swelling at T3-T6 segments and a central high signal area in the both halves of the spinal cord (Fig 1). Moreover, a post-contrast T1 weighted image showed mild heterogeneous patchy enhancement in the involved areas (Fig. 2). The patient was evaluated systematically for all probable causes of her symptoms, and finally, due to having a positive nasopharyngeal PCR test, she was considered to suffer from post-COVID-19 coronavirus transverse myelitis. She was treated with intravenous-immunoglobulin 0.4 g/Kg/day for 5 days, pulse of methylprednisolone 30 mg/Kg for 3 days, and other supportive cares without obvious results. Therefore, she underwent seven sessions of plasma exchange, which only had slight effects on her lower limb muscle strength on day 16. 

## Discussion

The focal inflammation and injury of the spinal cord, otherwise known as transverse myelitis, have a wide array of potential etiologies. Transverse myelitis has been well documented to be the result of viral and bacterial infections. It can also be the first sign of neurologic conditions such as neuromyelitis optica and multiple sclerosis, or can be associated with some immune system disorders such as lupus or sarcoidosis (6). However, near 60% of cases may remain idiopathic, suggesting that exact pathophysiology of the disease is unclear and may vary by etiology (7). Previous reports suggested that certain coronavirus genotypes including SARS-CoV in 2003 had neurotropic properties and may cause encephalomyelitis, Guillain-Barre, seizure, and loss of neurofunctional status (8). The first case of SARS-CoV-2 causing acute myelitis was published on March, 2020, from Wuhan, China, which was followed by a series of universal reports (3, 4, 6, and 8). Nevertheless, to our knowledge, this is the first report of TM in the field of pediatrics occurring after COVID-19. Thus, it should be noted that children can present with some severe types of COVID-19. 

Zhao and colleagues reported an association between COVID-19 and Guillain-Barre syndrome where neurological symptoms occurred without any preceding respiratory symptoms, suggesting a para-infectious process. It is still unclear whether TM occurs as a result of a direct impact of viral infection or as an autoimmune phenomenon. Moreover, it is still unclear whether the virus impacts vascular cells or disseminates into the nervous system via olfactory bulb when acting directly (4). Immune damage and cytokines by inflammatory storms at the early stage of SARS-CoV-2 infection might explain why the spinal cord is implicated at this setting (3). Furthermore, in advanced stages of the disease, neurological findings could be due to the effect of hypoxia sequels as well as respiratory and metabolic acidosis (4). However, the precise mechanism of the disease is unclear, and like in most post-infectious TM, an immune-mediated pathogenesis may be involved (8). We believe our patient was not involved in a cytokine storm status due to good CRP, IL-6 and Ferritin levels. Albeit, we cannot certainly consider whether the patient had a direct viral neurological impact or a delayed immunity phenomenon were happened, just like what happens in multi system inflammatory of childhood (MIS-C). We do not have PCR from CSF or some auto-antibodies as complementary parameters, and this appears to be the first pediatric post-coronavirus TM report. The patient’s medical file could be further investigated for NOM and other possibilities that we could not exclude despite our comprehensive systematic evaluation.
